# Pregnant Agencies: Movement and Participation in Maternal–Fetal Interactions

**DOI:** 10.3389/fpsyg.2020.01977

**Published:** 2020-08-14

**Authors:** Alejandra Martínez Quintero, Hanne De Jaegher

**Affiliations:** ^1^IAS-Research Centre for Life, Mind and Society, Department of Philosophy, University of the Basque Country, Bilbao, Spain; ^2^ChatLab, School of Psychology, University of Sussex, Brighton, United Kingdom

**Keywords:** pregnancy, participation, enaction, sensorimotor agency, embodiment, phenomenology of pregnancy

## Abstract

Pregnancy presents some interesting challenges for the philosophy of embodied cognition. Mother and fetus are generally considered to be passive during pregnancy, both individually and in their relation. In this paper, we use the enactive operational concepts of autonomy, agency, individuation, and participation to examine the relation between mother and fetus *in utero*. Based on biological, physiological, and phenomenological research, we explore the emergence of agentive capacities in embryo and fetus, as well as how maternal agency changes as pregnancy advances. We show that qualitatively different kinds of agency have their beginnings already *in utero*, and to what extent fetal and maternal movement modulate affectivity and individuation in pregnancy. We thus propose that mother and fetus are both agents who participate in pregnancy. Pregnancy then emerges as a relational developmental organization that anchors and holds its developing participants. We end the paper with reflections on ethical implications of this proposal, and suggestions for future research.

## Introduction

Pregnancy presents some interesting challenges to the philosophy of embodied cognition. Recently, in a project on the metaphysics of pregnancy, Kingma (2019) has pulled apart two options for conceiving of the relation between fetus and maternal body. Either the fetus is merely contained within the maternal body, or it is a part of the maternal body. In the first case, the so-called container model, the fetus is like a bun-in-the-oven or a tenant to its niche (Smith and Brogaard, 2003). Kingma rejects this view. While it is widespread, she argues it is philosophically hard to maintain. Instead, Kingma argues for the second option, where the relation between mother and fetus is considered a part-whole configuration. For instance, the maternal body functionally and metabolically integrates the fetus, and both collaborate on maintaining the pregnancy. Kingma finds this view metaphysically more interesting, and more in line with biological and physiological knowledge of the process of pregnancy. But even accepting it, it “remains poorly understood” how far and in what ways each of them participates in this kind of relationship (Kingma, 2019, p. 626).

While we take Kingma’s metaphysical lay-of-the-land as general stage-setting for our arguments, her analytical approach also has some limitations. It shares with the container model a rather static account of pregnancy. Both overlook the fact that gestational conditions change and that the kinds of interactions that blastocyst, embryo, and fetus have with the maternal body differ greatly. In this sense, these models downplay the different kinds of interactions that take place throughout the gestational process.

Phenomenological insights provide interesting approaches to the interactions in pregnancy (Young, 2005; Smith, 2016; Moran, 2017). We will here follow and extend Jane Lymer’s (2011) proposal that mother and fetus maintain a bidirectional affective-communicative relationship. By this, she means that maternal movement and affect guide or imprint on the fetus’s ways of moving and being. Lymer connects maternal experience with empirical studies that show fetal responses to maternal actions, like voice, touch (Marx and Nagy, 2015), and stress situations (DiPietro et al., 2013). This combination of phenomenological and empirical research provides an experiential and existential advance on the analytical question of whether the fetus is merely contained within, or rather a part of its mother.

In this paper, we aim to further investigate the relationship between fetus and mother. To flesh out what this relation is, we will rely on biological, physiological, and phenomenological research, and suggest a way to operationalize maternal–fetal interactions. This allows us to elucidate pregnancy as a phenomenon of developmental relationality. For this, we will study pregnancy under two questions: *To what extent are fetus and mother agents?* And, *to what extent do they participate in relation with each other?* In asking these questions, we investigate the beginnings of both agency and participation in pregnancy. As living beings, we assume fetus and mother both have stakes in their own being and in their relation.^1^

Embodied views support the idea that prenatal bodily interactions provide the necessary preconditions for human cognitive development (Gallagher, 2005; Delafield-Butt and Gangopadhyay, 2013; Fuchs, 2018; Ciaunica and Crucianelli, 2019). Within embodied approaches, enactive researchers in the Varela–Thompson–Di Paolo tradition take further steps, by explicitly taking a life-mind continuity view. On this approach, mind begins with the processes of living (Varela et al., 1991; Weber and Varela, 2002; Di Paolo, 2005; De Jaegher and Di Paolo, 2007; Thompson, 2007; Di Paolo et al., 2010; Di Paolo and Thompson, 2014). Minimal living beings, such as single-cellular organisms, already are minimal sense-makers on this view. Operational definitions of sensorimotor and biological agency provide grip on this idea. In this paper, we take this enactive perspective.

The analysis of pregnancy we perform here tests the limits of the enactive view. In quite a literal sense, central concepts of the enactive approach, such as autonomy, agency, and sense-making, come into existence in pregnancy. Studying movement in pregnancy can elucidate the developmental beginnings of sensorimotor agency, and provide a view that places these beginnings earlier than has been proposed in enactive theory so far (Di Paolo et al., 2017).

As Di Paolo (2018, 2020) has recently suggested, the enactive approach benefits from being expanded with Gilbert Simondon’s idea of *individuation*. With this idea, Simondon processualizes the notion of the individual (Simondon, 2005). Thinking of fetus—and mother—as individuat*ing* may seem intuitive enough. But we do not only mean by this that they are both ongoingly developing as individuals. We also mean to refer, with Simondon, to their ontology as self-individuating beings. Self-individuation means that living beings avoid full stability (which would correspond to death) by ongoingly renewing metastable states rich in potentialities. That is, as they build themselves, living beings also build themselves *out of* their material and energetic environment. And as they produce themselves, they distinguish themselves from their environment. In enactive terms, living beings both self-produce and self-distinguish (Di Paolo et al., 2017; Di Paolo, 2018). Self-production and self-distinction are opposing tendencies between which living beings continually dialectically navigate a course of life. This idea, which we explain further in the next section, forms the basis of our analysis of pregnancy.

These conceptual innovations are reflected in our terminology. We use the terms fetus and mother, maternal body, maternal organism, and maternal person to refer to those who take part in and together make up pregnancy—the participants of pregnancy.^2^ Pregnancy itself, we will show, constitutes a new relational organization. This means that pregnancy is a particular relational process, which has particular implications for both maternal and fetal agency. Among these implications are that both fetus and mother develop and individuate not only in relation to each other but also to pregnancy itself *as* a relational organization. It is in this sense that we will defend that the relational process of pregnancy anchors and holds the fetus and mother. Therefore, in this paper, we take pregnancy as an emergent relational organization, with mother and fetus as its active participants.

The argument of the paper proceeds in four parts. We first introduce the operational enactive concepts of autonomy and agency, together with the Simondonian idea of individuation. Then, applying these concepts, we show how agency emerges in embryogenesis, in an analysis of how embryo and maternal body coordinate in the process of implantation. Then, we explore fetal sensorimotor agency. Finally, we show how fetus and mother modulate their cognitive-affective experiences in touch and movement, and how mother and fetus participate in the relational development that is pregnancy. Our intention is not to give an exhaustive description of agency in every stage of the pregnant process, but rather to highlight and specify it in a few developmental milestones across pregnancy: at implantation, and in the first developments of fetal movement and touch. We conclude with the idea that pregnancy is “not one, not two” (Varela, 1976), meaning that pregnancy is a level of organization that constitutes—as such—a new individuating process, in which its participants relate and all elements of which co-constitute each other. We close the paper with some ethical considerations regarding agency that may be addressed in future research and provide some suggestions for further empirical questions throughout.

## Enactive Concepts

The enactive approach explains how movement and agency are not only individually guided but develop in participation with others (Varela et al., 1991; De Jaegher and Di Paolo, 2007; Thompson, 2007; Di Paolo, 2016). Enaction understands development as an ongoing process of self-production and self-distinction (Di Paolo, 2019). This means that when a cognitive system differentiates itself, an associated milieu emerges with it at the same time: “[cognitive systems] enact a world as a domain of distinctions that is inseparable from the structure embodied by the cognitive system” (Varela et al., 1991: p. 140). Most cognitive systems not only produce and individuate themselves but can also regulate their interaction with the environment. This is what we call agency. Here, we introduce the enactive operational concepts of autonomy and agency, enriched with Simondon’s notion of individuation. Looking at pregnancy from the perspective of this conceptual coalition will allow us to bring to light elements of agency and participation in pregnancy that have remained hidden until now.

### Autonomy

The enactive approach is largely built on the biological concept of autonomy (for a systematic review, see Moreno and Mossio, 2015). In this context, autonomy is the capacity of a system to produce and maintain the processes that constitute it as a system. Autopoietic systems (a particular kind of autonomous system) self-organize in the sense that they are networks of mutually enabling relations—mainly biochemical processes of exchanging matter and energy (Maturana and Varela, 1980). Metabolism is the best example of an autonomous process in living systems. In metabolism, products from a set of reactions reincorporate into the system, as the basis for a next reaction, in such a way that products become processes. Autonomy in metabolism has two fundamental yet opposing tendencies: to keep thermodynamically open but operationally closed. That is, to let in flows of matter and energy as they are needed for regeneration, growth, or to fuel activity; but the system also tends—and needs—to avoid the tendencies that would lead to decay and indistinction from its environment, and so to close itself to some perturbations. This makes for a primordial tension between self-production (openness) and self-distinction (closing) (Di Paolo et al., 2017; Di Paolo, 2018). Autonomy allows us to see how life dynamically self-organizes.

### Precariousness, Adaptivity, and Sense-Making

The operational concept of autonomy, however, is not enough to describe the differences between living and non-living systems. For this, Di Paolo (2005) has proposed the concepts of precariousness and adaptivity. Organisms are precarious not in the first place because they decay, but because their individuation involves the tension between self-production and self-distinction. All far-from-equilibrium systems tend to decay, but only living systems actively operate to counteract dissipation by navigating this tension. Thus self-individuation has an intrinsic dialectic that maintains the system in a constant turn-over: from self-distinction to self-production and back again. Neither self-distinction nor self-production are viable on their own: too much of one would destroy the other. The tension between them needs to be ongoingly solved (it is never finally resolved), by taking action.

In this sense, autopoiesis is full of potential, as it dialectically leads the system to a further step: to relate to its own existence and the surrounding elements. Di Paolo (2005) proposes to understand this as the autonomous system’s adaptivity, a necessary step to pass from mere physico-chemical interactions to a veritable perspective on the world. The living being can be said to be concerned with its existence (Jonas, 1966) and endowed with a sensitivity to discriminate between different states, recognizing when it approaches the boundaries of its zone of viability, and able to avert tendencies that would result in crossing this boundary. In this sense, the more adaptive an organism is at any stage in its life, the more potential for agency it has.

It is also here that sense-making begins. Sense-making is the enactive way of describing cognition in general. It does not immediately imply a sophisticated cognition or a distinction between cognition and affect, but first of all a primordial sensitivity that is affectively constituted in interaction with the organism’s environment. An adaptive organism is meaningfully affected by its interactions with the world, and so establishes the norms by which it evaluates or discerns these interactions, from the organism’s perspective as embodied and situated in its world (Colombetti, 2014).

### Agency

**FIGURE 1:**
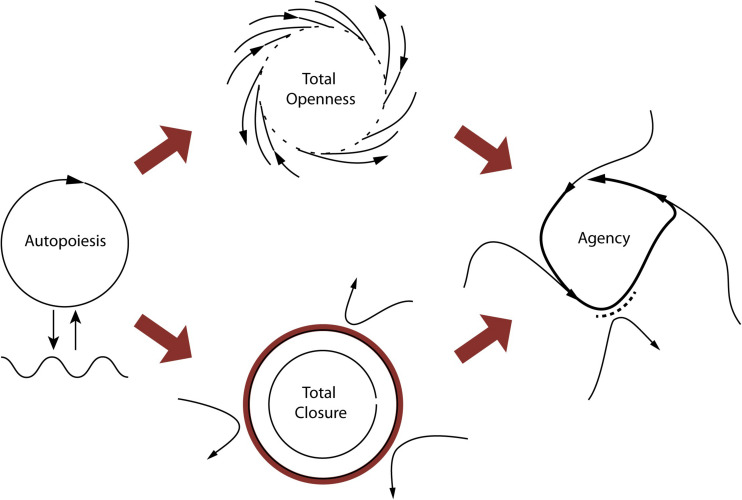
The primordial tension of self-individuation. On the left, a self-enclosing circle represents the condition of operational closure, and the autopoietic system’s coupling with the environment. The ideal realization of the condition of self-production is shown at the top center, where the arrows represent material/energetic flows in the environment. In this ideal case, all of the environmental flows contribute to producing the system. The ideal conditions for self-distinction demand the opposite situation (bottom center), which would be satisfied by building an impenetrable barrier, preventing any environmental flow from affecting the system. In both cases, actual self-individuation is impossible (this is depicted by the open circles). The tension between the two requirements is overcome by managing their divergences over time, through adaptive, asymmetrical regulation of the coupling with the environment, accepting certain environmental flows and rejecting others (figure on the right). A system able to manage these inherent tensions in material self-individuation is an agent according to our definition. Copyright 2017 Ezequiel A. Di Paolo, T. Buhrmann and X. Barandiaran, with permission.

Agency adds to autopoiesis and adaptivity the capability of an organism not only to interact but to regulate its interactions with its associated milieu, already specified by the process of self-individuation (see Figure 1). As autopoietic, the organism self-maintains, but mere interactions with its environment do not allow it to counteract environmental conditions if needed. In becoming adaptive, the organism self-maintains, *and* its interactions are now sensitive to changes in the environment, so it adapts internal constraints to them. Further, as an *agent*, the organism displays world-involving action: an agent acts upon external constraints by regulating its interaction with the environment. It forms a minimal ‘perspective’ on the world (at the very least in terms of “good” or “bad” for self-maintenance), which opens up its sense-making (as sensitivity to what can fulfill its needs or circumvent its constraints). The notions of individuality, asymmetry, and normativity capture in more detail the conditions for agency, so we will explore them next (Barandiaran et al., 2009; Di Paolo et al., 2017).

#### Individuality—Individuation

As an individual-in-becoming, a system distinguishes itself from its immediate surroundings. To have an intuitive understanding of self-individuation, imagine that the system ‘encapsulates’ its constitutive parts into a functional or physical boundary.^3^ In organizational terms, this is defined as operational closure (Varela, 1979; Di Paolo, 2009; Di Paolo and Thompson, 2014). This means, as in the definition of autonomy, that the boundary between system and environment is not externally given, but constituted by its ongoing processes of self-organization.

But the notion of individuality, as the first condition for agency, is problematic in the case of pregnancy (see Griesemer, 2018 for a review). There is no agreement on the stage at which we can consider the developing organism a biological individual. Nuño de la Rosa (2010), for instance, has argued that embryos are not biological individuals until organogenesis is complete and they reach functional and structural integration. A deeper exploration of individuation, then, must go beyond the idea of encapsulation, and refine the idea of operational closure, in favor of a processual approach that considers the successive transformations over the life span (Simondon, 2005; Di Paolo, 2018, 2020).

We consider that Simondon’s idea of *individuation* precisely emphasizes the open-ended process of development better than the notion of individuality. Individuation captures moment by moment the process that continually specifies its own domain of relations that constitutes itself and its environment. We also propose that the Simondonian notion of *individuation* grounds the process of becoming (following Di Paolo, 2018, 2020), even before functional integration is achieved during gestation. Taking this enactive-Simondonian approach, we emphasize the primordial requirement for agency—individuation—as an open-ended process.

#### Asymmetry

The next condition for agency is interactional asymmetry, or the ability to modulate at least some of the interactions with the environment. As Di Paolo et al. (2017) point out, agency implies that the exchange between the organism and the environment is not equal. If it were, the exchange conditions would be an even and unconstrained flux of matter and energy between agent and environment, and the existence of the organism would depend only on the external enabling conditions. In such a case, a system would not self-distinguish but would dissipate when the external conditions are depleted. In contrast, agency accounts for the adaptive powers that counteract environmental threats by acting upon external constraints. Living beings have adaptive capacities that allow them to do this. In interactional asymmetry, the organism shows a world-involving action by externalizing its activity. Bacterial chemotaxis or chemical signaling are minimal examples of this.

#### Normativity

The third requirement of agency, normativity, goes back to autonomy: the norms upon which the agent is acting must be established by the system itself. Normativity is implicit in the autonomous living organization. An autonomous, self-distinguishing and self-producing entity produces its own vital norms (Thompson, 2007). According to these intrinsic norms, actions contribute to the maintenance of the system or put it at risk. If the norms were to be externally given, then the system would be heteronomous. Normativity in minimal agents refers only to the norms that help to keep the system away from disintegration. In biological agents, it minimally means to keep alive by following metabolic norms. But in more complex living systems, autonomy can expand, producing additional normative frames associated with different domains (biological, sensorimotor, intersubjective, linguistic, etc.). These new normativities can be partially decoupled from metabolic norms and might even enter into conflict with them or with other normative levels.

In short, agency expands the autonomous capacities of a living being. As we said, agency is potential in every living system and emerges when the tension between self-distinction and self-production reaches a critical point, from which it unfolds (or which it, again and again, reintegrates). Expanding the scope of autonomy, then, is expressed in new ways of mediating and regulating its relationship with the environment. This is an important point in our analysis of the maternal–fetal relationship. We will see that, as fetal organization moves into new phases of individuation, it remains rooted in a fundamental process of autopoiesis, renewing potentialities, generating new levels of interiority and, with them, expanding the scope of agency and the capacities to interact with the environment.

## Beginnings of Agency and Participation in Pregnancy

Now, applying these concepts and definitions to pregnancy, what is agency in the maternal–fetal relation? Starting from the enactive concepts, we realize that even in the most basic forms of biological organization, organisms will—at least in some moments—display some kind of agency. To illustrate the emergence of agency in pregnancy, we will study the case of implantation. The implantation process, we propose, creates the tensions that mark the beginnings of self-individuation for the embryo.

### Fertilization

First, let us consider whether the female egg is a minimal autopoietic organization. When released from the ovaries, the human ovum is a free-living cell, covered by an extracellular matrix called the zona pellucida. *In vitro* studies show that metabolic activity in the oocyte is low because its mitochondria are still immature (Lubis and Halim, 2018). Thus, one might argue that the oocyte is not capable of autopoiesis (self-production and self-maintenance). But we consider this minimal metabolic activity to be sufficient to attribute autopoiesis because the female egg produces enough energy from oxidative phosphorylation metabolism to endure at least 12–24 hours (Lubis and Halim, 2018).^4^

After ovulation, the uterine finger-like structures, called *fimbria*, catch the oocyte and guide it into the fallopian tubes (Lyons et al., 2006). If fertilization occurs within 12–24 hours, the egg incorporates the sperm’s genome, creating the first primordial tensions in the zygote; a diploid cell that will rapidly enter into cleavage. During cleavage, the egg and sperm’s pronuclei fuse, and the zygote starts mitotic division. It divides into two, then four, and when it reaches the 8-cell stage, the embryonic genome starts to activate, increasing metabolic activity on its own (Lubis and Halim, 2018). The system slowly starts to depend less on the maternal gene expression and more on the embryonic genome (Lubis and Halim, 2018).

**FIGURE 2:**
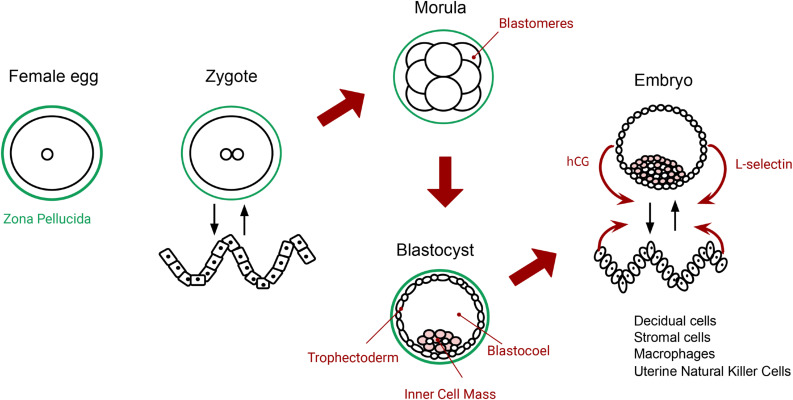
The primordial tension of self-individuation, from fertilization to implantation. The zygote (autopoietic system) realizes self-production (metabolism) and self-distinction (it maintains the membrane and the system distinct from the environment). The wavy pattern on the left-hand side depicts endometrial cells before differentiation (an environment for the zygote). The morula (at the top middle) moves toward more openness (self-production), expressed by the pluripotential blastomeres. Then, the blastocyst pulls again into self-distinction, or closure, when it produces a new boundary: the outer layer of cells or trophectoderm (at the bottom middle). At implantation, blastocyst and endometrial cells change their morphology and gene expression (both show adaptivity) and turn into a functional embryo and receptive decidual cells. The way they coordinate with each other (interactional asymmetry) opens the possibility to adhere, attach and ‘invade.’ Both sides interact asymmetrically at the local level: they detect, modulate and respond according to each other’s activity (curved red arrows). The initial potentialities in the zygote (autopoiesis) are fully expressed in the blastocyst by more adaptive regulatory capacities (biological agency), depicted on the right side.

Cleavage continues until it forms a ball of 32 cells called blastomeres. Blastomeres are pluripotential cells, fully open to becoming any possible cell type. We propose to take this new multicellular state called the *morula* (top middle of Figure 2) as a concrete example of the ideal self-production, illustrated in Figure 1. The full potential present in every blastomere illustrates the openness of self-production. In this sense, the blastomeres make the morula a highly unspecified system (not an individual), as it is poorly differentiated, yet full of potentials.^5^

With an increase in metabolic activity, the embryo then enters into blastulation. We identify this process with self-distinction (bottom middle drawing in Figure 2), which corresponds to ideal closure in Di Paolo et al.’s conceptualization (Figure 1). To self-distinguish, a group of the cells in the system starts to form a new membrane, called the trophectoderm. The metabolic activity in the trophectoderm increases glucose uptake and introduces oxygen into the system. This creates a fluid-filled cavity inside the system called the blastocoel (Lubis and Halim, 2018). The rest of the cells will start to proliferate and compact to form the inner cell mass (illustrated as pink cells in Figure 2). When these three structures—trophectoderm, inner cell mass, and blastocoel—are mature, the blastula hatches from the zona pellucida. Now we have a new differentiated system which has produced its own new physical boundary. Note that in terms of self-individuation, this is a move toward more differentiation, but never “total” closure. Then uterine contractions, along with the uterus’s cilia, lead the blastocyst toward the endometrial implantation zone (Lyons et al., 2006).

### Implantation

It is here, we argue, that biological agency emerges in pregnancy. Rather than a free and unconstrained exchange of chemicals in the ideal autopoietic cell, implantation shows highly specific and active co-regulation by embryo and endometrium. To show why and how agency emerges at implantation, we discuss evolutionary, clinical and biological evidence that challenges passive views of implantation.

Nuño de la Rosa et al. (2019) provide an evolutionary developmental account that conceives of implantation as a participatory process. Their proposal contrasts with traditional pictures of “the maternal–fetal conflict” in which the embryo aggressively invades, takes control of maternal immunity and exploits maternal resources against her interest (Ashary et al., 2018). Against such views, Nuño de la Rosa et al. (2019) propose that pregnancy is the result of evolutionary and developmental co-adaptation—a historical process through which maternal organisms and embryos co-evolved in Eutherian mammals. They explain that implantation is an inflammatory process of the endometrium, but in humans—Eutherian mammals—this process is particularly invasive. The authors suggest that invasiveness likely evolved as a result of both embryo and mother taking advantage and control over the inflammatory process (Nuño de la Rosa et al., 2019). They emphasize that the innovation (even a major transition in evolution) lies in this relational core of the maternal–fetal unit, and not only in the placenta as is widely accepted. Thus, human pregnancy demands both fetus and maternal organism to display specific adaptive strategies, enabling greater invasiveness and longer pregnancies in Eutherians, compared with other mammals (Wagner et al., 2014).

According to clinical and *in vitro* studies, implantation forms one of the greatest challenges to setting up pregnancy. Clinical studies report that around 75% of pregnancy losses occur at this stage (Norwitz et al., 2001; Cha et al., 2012).^6^ Failure can be attributed either to genetic abnormalities in embryo or to impaired differentiation in decidual endometrial cells (Norwitz et al., 2001; Cha et al., 2012). Such defects would impede blastocyst adhesion and attachment. Even if both sides are fully functional, they have a very restricted time span—the implantation window—to coordinate their activity (Cha et al., 2012). On the one hand, if they implant outside of this time window, implantation will be shallow or defective (Cha et al., 2012). On the other hand, if blastocyst and endometrial cells fail to coordinate, the blastocyst will not attach and die (Norwitz et al., 2001). More even, the maternal immune system might attack the blastocyst if it is detected as deleterious and non-viable (Ashary et al., 2018). Thus, implantation requires a competent embryo and a receptive endometrium to create the conditions for implantation (Norwitz et al., 2001; Teklenburg et al., 2010; Cha et al., 2012). It is in this sense that embryo and maternal body have adaptive and self-regulatory capacities to counteract the constraints of time, immune response and gene under-expression. Agential capacities at implantation are crucial for the viability of pregnancy.

Thus, based on biological knowledge, we can describe *how* implantation marks the beginnings of biological agency. Both blastocyst and endometrial tissues actively regulate gene expression, transcription factors, signaling pathways, inhibiting factors, and growth factors during the implantation process. For instance, the blastocyst secretes human chorionic gonadotropin hormones (hCG) that remove anti-adhesive factors in the endometrial epithelium. Next, the embryo uses the receptor L-selectin to facilitate its adhesion. This allows the blastocyst to roll over the epithelium, and sense a receptive place for implantation (Ashary et al., 2018). However, this mechanism is too weak to adhere and implant, thus it requires that the endometrial epithelium also displays specific adaptive capacities. The endometrial tissue self-modifies some of its cells, through differentiation, into specific types that support implantation: namely decidual stromal cells, uterine natural killer cells, and macrophages (Nuño de la Rosa et al., 2019) (see Figure 2). In turn, the embryo needs to coordinate with these specialized cells within the implantation window. Once the embryo implants, the maternal organism needs to adapt her main physiological systems to ‘integrate’ it to her homeostatic processes. After implantation, maternal nervous, cardiovascular, locomotor, and immune systems will accommodate to ‘hold’ the embryo (Nuño de la Rosa et al., 2019). In this picture, agency emerges from the relation between embryo and maternal environment at two levels: *local* tissues involved in implantation and *global* reaccommodation of the whole maternal organism.

In sum, the viability of implantation—and thus of pregnancy—depends on the adaptive capacities of the embryo and on maternal local and global adaptations. We propose that biological agency emerges here, from the need to establish coordinated activity by adaptations on both sides (drawing on the right-hand side of Figure 2). Embryonic individuation may indeed be cradled asymmetrically here^7^ (it is highly supported by the pregnant organism), but this does not lessen the fact that what is going on is individuation and the beginnings of agency. Indeed, it is not clear whose activity is more determinant for implantation, as empirical studies *in vivo* for humans are limited, and the underlying molecular mechanisms are not well known (Teklenburg et al., 2010).

This detailed description of implantation serves to illustrate the abstract concepts of autopoiesis, agency, and individuation (see description at Figure 2). At implantation, the embryo interacts with the uterine cells and meets the three requirements for agency: it reaches a certain degree of individuation producing its own boundary; it acts against decay upon external constraints of uterine signaling (asymmetry); and acts according to the norms of its metabolism and gene expression (normativity).^8^ Thus, pregnancy helps us to understand, refine and specify the fundamental tensions of self-individuation. The enactive notions of autonomy, individuation, self-production and self-distinction are sometimes criticized for being rather abstract (though see, e.g., Di Paolo et al., 2017), but we can show, by studying the physiological details of pregnancy, how agency emerges from the tensions between self-production and self-distinction.

### Pregnancy as an Emergent Relational Organization

In pregnancy, two individuating organisms—each dealing with their own ongoing tensions between self-production and self-distinction—grasp into each other, literally and metaphorically. Their processes of individuation emerge in dependence upon each other. Furthermore, they are also dependent on the intertwined process itself that emerges between them. Thus, pregnancy is an emergent relational process. We propose that pregnancy individuates the developmental relation between maternal body and embryo. This relation can “solve”—always temporarily, again and again in new configurations—the tensions between the maternal and fetal individuations, and “hold” them as they do so. By this, we mean that both depend on this relation for their existence as long as the relation holds. In doing so, the biological relational organization anchors them to the process of pregnancy. All three elements—maternal and fetal individuations, and their relation—together make pregnancy possible. This is particularly true at the time of implantation. Conceptualizing pregnancy as this emergent relation full of potentialities and tensions is the fundamental step that we consider necessary for any analysis of pregnancy. It drives us to move away from an understanding of pregnancy focused on two already individuated systems—and how they relate—toward understanding what goes on in a dialectical-relational way. As pregnancy self-organizes, it constitutes, but does not over-determine, each of the individuation processes of maternal body and fetus.^9^

From here, we now move on to fetal development. We will see how the scope of agency changes again with the beginning of the first movements in the fetus. While the biological relation that grounds pregnancy holds metabolic needs in both, the potentialities in the embryo now create room to unfold actions that may not contribute directly to metabolism, but to regulating interbodily space.

## Sensorimotor Agency in Fetus

Now that we have traced the emergence of biological agency in early pregnancy, we take the next step and examine the development of sensorimotor agency. Contrary to the widespread view of fetal movement as chaotic, limited and constrained, we suggest that fetuses unfold complex sensorimotor capacities. This involves an expansion in the scope of fetal autonomy and a new relation with the mother. To show this transition, we begin with the emergence of general movements and startles in the fetus, and then discuss breathing, suckling, and swallowing movements, and their integration *in utero*. Finally, we look at fetal touch, which expands the scope of agency toward self-affection and intercorporeity. The aim of this section is twofold: to show that fetal sensorimotor capacities are relational and situated in pregnancy, and that the fetus is a sensorimotor agent-in-becoming.

In contrast with biological agency, sensorimotor agency is the capacity of an organism to regulate its interactions with the environment by coordinating sensory and motor capacities. In line with Di Paolo et al. (2017), we use the term “sensorimotor regularities” to describe the predictable variations between sensory stimulation, neural, and motor activity (Di Paolo et al., 2017, p. 43). These sensorimotor regularities can be classified into different kinds, but we are only going to refer to sensorimotor *coordination* and sensorimotor *schemes*. The former are organized patterns of activity (and the basic units of our analysis)^10^ from which the latter emerge. Just like an autonomous system, a sensorimotor scheme dynamically organizes different sensorimotor coordinations, so it forms an operational closure in an adaptive, self-regulated way. As an autonomous system, the sensorimotor scheme must function according to its intrinsic normativity, as it puts together more or less successfully (fluently, efficiently, etc.) different sensorimotor patterns to achieve a goal (see Di Paolo et al., 2017).

### General Movements

Dynamical approaches to locomotor development suggest that even the simplest movement can be considered a sensorimotor event and self-organized (Thelen and Smith, 1994). Studies in fetal movement, in particular, have shown how first movements emerge in the fetal body from the self-organization between its early nervous system, the fetal body support structures (muscles, bones, and organs), and elements of the uterine environment (fluid density, structural support, pressure, available space, etc., see Smotherman and Robinson, 1988; Mori and Kuniyoshi, 2010). For instance, Piontelli (2010, 2015) explains in two recent reviews that at the very beginning of the fetal stage the motor cortex—the area of the cortex that in adults is thought to control movement—is not yet developed. Thus, between weeks 7 and 13 most movements are produced from the central pattern generator in the early spinal cord and immediately enter into different loops of motor and sensory coordination.^11^

General movements are a clear example of how first fetal movements emerge from self-organization phenomena. Around week 8, the central pattern generator produces abrupt, shock-like jerks of the entire fetal body. These so-called startles appear to set in motion general movements, as they displace the entire fetal body in a pronounced upward thrust, provoking the limbs or head to shift position (Piontelli, 2010). As Piontelli remarks, at this stage limbs are weightless with respect to amniotic fluid density and, as such, they are “relatively ‘light’ and easily shifted” (Piontelli, 2010, p. 23). In turn, such movement of arms or legs often triggers another counter-reactive movement. These general motions can induce a completely new pattern in a previously motionless limb. In Piontelli’s words: “for instance, a hand may start to touch the face, or a leg may change its position, be flexed or extended, or both” (2010, p. 23). Sensory feedback gives the possibility to react to these motions, and eventually to adjust them. While the fetus is moved by the startle, it becomes progressively sensitive to itself and the different elements of its environment.

This suggests that self-organization in fetal movement starts as a biomechanical rearrangement and progressively enriches sensitive and regulatory capacities. Fetuses might find with startles that a hand is movable; moving the hand they might feel that touching the face with the hand is more sensitive than touching the umbilical cord or the uterine wall. As Piontelli points out, “through general movements fetuses begin to ‘learn’ to move and to attune their motions” (Piontelli, 2015, p. 128). As we will show, sensorimotor coordinations move from mere self-organizing motor patterns to more adaptive, self-regulated patterns of movement that sustain different sensorimotor schemes.

### Breathing Movements

One of the most consistent sensorimotor coordinations is fetal breathing movement. Fetal breathing movements are detected around week 10 and differ from aerial breathing (Piontelli, 2010; Fraga and Guttentag, 2012). Unlike newborns’ aerial respiration, where the lungs support gas exchange, in the fetus the placenta is the main oxygen supplier. Instead of air, fetal lungs are filled with a fluid produced by the lungs’ epithelium. Fetal lung fluid constantly expands the lungs and increases lung density and pressure (Piontelli, 2010).

We said that fetal breathing is a sensorimotor coordination because it self-organizes movements of expansion and contraction between the diaphragm, the chest and the abdomen (Piontelli, 2010). Among chest and diaphragmatic movements, breathing movements regulate glottis dilation to ease the outflow of lung liquid and release pressure. Diaphragmatic contractions also control glottis aperture to limit the amount of liquid that flows out of the lungs (Wallace et al., 2015). This coordination is important because if lung liquid density is too low, it produces pulmonary hypoplasia, and in severe cases, alveolar collapse. On the other hand, if lung liquid volume is too high—during the absence of fetal breathing movements (i.e., apnoea periods)— it unbalances intra-pulmonary pressure (Wallace et al., 2015). To maintain optimal intra-pulmonary pressure, the ratio between amniotic liquid and lung pressure should be close to zero. However, in cases of underdeveloped lungs, higher lung pressure can help to accelerate lung growth and maturation (Wallace et al., 2015). Likely additional functions of fetal breathing movement are to prevent asphyxia and to prevent the amniotic fluid from reaching the lungs and causing damage (Piontelli, 2010).

It is worth mentioning that there are some conceptual problems with the interpretation of empirical findings in fetal breathing movements. First, there is no agreement about their function. Second, because fetal breathing movement does not attempt to bring oxygen into the lungs, received developmental views hypothesize that they might be a preparatory stage for the ‘real’ function in the newborn. Such views thus focus on the ‘grown’ individual. This kind of teleological explanation has been extensively criticized by the organizational approach to biological functions (Mossio et al., 2009), and by the idea of development as the retroactive realization of situated potential (Maclaren, 2017). It is how *we*, as external observers, *know* that these movements will eventually contribute to aerial respiration.

In contrast, under an enactive point of view, fetal breathing movements can be explained by their contribution to the actual system. When observing these movements, we see that the fetus actively regulates lung density by producing lung liquid and accommodating intrapulmonary and amniotic pressures. Also, the fetus does not realize breathing movements in a vacuum. Amniotic composition and pressure co-vary with maternal metabolism, movement and clinical conditions (Wallace et al., 2015). For instance, caffeine or some medications like amphetamines increase rates of fetal breathing, while depressants of the nervous system like anesthetics, ethanol, and narcotics inhibit fetal breathing activity (Fraga and Guttentag, 2012). In this sense, fetal breathing movements can be constrained by the mother, but are actively sustained by the fetus. Indeed, in absence of fetal regulation, e.g., when the fetus is anesthetized, paralyzed or dead, the lungs rapidly lose their density (Wallace et al., 2015). Also, without practising expansion and contraction movements, later on the newborn’s lungs would collapse upon taking the first puff of air (Piontelli, 2015; Wallace et al., 2015). Thus, from an enactive perspective, fetal breathing movements enable, rather than predetermine neonatal breathing.

According to these descriptions, fetal breathing movements can be considered an emerging sensorimotor coordination as they: (1), produce mutually enabling conditions, (2), define themselves as a system separated from, yet interacting with other systems, and (3) modulate their relation with the medium by equilibrating—accommodating and assimilating—fluid density, space, and pressure. While fetal breathing movements ongoingly solve these tensions between the amniotic liquid and intrapulmonary density, they form a sensorimotor coordination through adaptive and self-regulated patterns.

### Swallowing

Fetal breathing movements are connected with other sensorimotor coordinations like swallowing. Indeed, the fetus swallows part of the lung liquid released during breathing. Swallowing requires the palate to fuse, to separate the vocal and nasal cavities. This happens around week 10 (Piontelli, 2015).^12^ Proper swallowing prevents the fluid from going into the lungs, and ensures that the liquid taken in remains in the stomach. At the very beginning, swallowing movements are not directed or controlled, but as pregnancy advances many muscles (about 24) and cranial nerves (6) start to regulate the swallowing cycle (Piontelli, 2015).

According to Piontelli (2015), the swallowing cycle self-organizes as follows. Tongue and mouth coordinate to draw amniotic liquid into the mouth; different tongue movements help to pass it through the esophagus to the stomach; the oesophageal sphincter closes if necessary to prevent chokes or reflux, so the liquid can be digested and, finally, fetal urine is released back into the amniotic fluid. Urine modifies the composition of the swallowed fluid, and then the cycle repeats.

As in the case of fetal breathing and lung development, the function of swallowing is not well known. Some researchers suggest that swallowing movements play a role in gastrointestinal development (Piontelli, 2010). But again, we insist that the actual swallowing anticipates no future neonatal gastrointestinal function, but enables it. Indeed, it is very likely that swallowing partially contributes to fetal nutrition—as 60–70 percent of protein is absorbed from the amniotic liquid. More importantly, swallowing also contributes to regulating the amount and composition of the amniotic liquid. Changes in its composition will alter the proportion or shape of fetal organs (Wallace et al., 2015). This may explain why the maintenance of the amniotic liquid seems to be increasingly taken over by the fetus to counteract a decrease in the amount of amniotic fluid.

Thus, the elements that compose swallowing movements are highly interdependent. An alteration in one aspect necessarily implies a re-accommodation and assimilation that will re-organize the whole swallowing pattern, or even a coordination between swallowing and breathing, e.g., releasing more lung liquid in the absence of urine. In this sense, breathing and swallowing are two sensorimotor coordinations that become more individuated, to such an extent that they organize into patterns that regulate fetal-uterine relations that are not strictly metabolic.

### Suckling

When swallowing and breathing stabilize and coordinate, suckling emerges as a new sensorimotor regulation. Suckling movements coordinate motions of the mouth, tongue, and lips to create a partial vacuum in the mouth that facilitates drawing liquid into the mouth (Piontelli, 2015). This requires more motor control of the muscles of the tongue, lips and mouth than that practised in early breathing: more regulated ‘inspiration’ movements to sip liquid and create the vacuum. Swallowing also needs to be more stabilized in form and rhythm to impede choking. Only when the fetus has stabilized different movements involved in breathing and swallowing, like gasping, mouthing, and closing the glottis, can it accomplish suckling. For this reason, it is one of the latest movements to be detected in pregnancy, between 34–36 weeks. Indeed, when neonates are born preterm, suckling is hardly coordinated and the newborn is not able to accomplish nutrition or breathing on its own (Piontelli, 2015).

### Fetal Movement Coordination

Based on the evidence discussed above, fetal movement appears to be highly organized. According to the enactive approach, this kind of organized movement can be taken as evidence for the origins of sensorimotor agency. Indeed, Di Paolo et al. (2017) address this same scheme in breastfeeding in newborns. According to them, breathing, swallowing, and suckling dynamically organize in such a way that they form an operational closure, achieving breastfeeding in an adaptive, self-regulated way. Their explanation is based on Piaget’s descriptions but adds a dynamical systems view on how both agent and environment enter into the equilibration process (Piaget, 1975; Di Paolo et al., 2017). As such, the sensorimotor scheme of breastfeeding consists in the organization of different sensorimotor regularities—suckling, swallowing and breathing—that assimilate new environmental aspects that were absent *in utero*: the nipple or bottle to suck, the milk to swallow, and the air to breathe. Sensorimotor agency, however, can already be observed *in utero*. Understanding agency as a relational process, we can trace the emergence of sensorimotor schemes to early pregnancy. As we will show, in the case of pregnancy, it is more evident how both agent and environment covary, mutually modify initial conditions, and participate in the process of self-organizing sensorimotor regularities.

In contrast, a more individual-centered approach might consider early fetal movements to be chaotic and ‘disorganized’ (Thelen and Smith, 1994; Piontelli, 2010). Thelen and Smith (1994) called these kinds of theories ‘adultist’ because they take the adult as the ultimate stage of a linear progression. In consequence, the actual capacities in embryo, fetus, infant or child are decontextualized and misinterpreted.^13^

### Fetal Touch and Affectivity

The ‘adultist approach’ also permeates the study of fetal perceptual capacities. Fetuses are often compared with newborns, and the differences in organization and the relational situation *in utero* are disregarded. For instance, there is a widespread belief that fetuses receive auditory signals like neonates do, and therefore, that maternal voice or music can improve cognitive capacities (Piontelli, 2015). Though this conclusion may be true, the most developed sensorial capacities in fetuses are the tactile and olfactory systems, not the visual or auditory ones. Thus it remains unclear to what extent fetal reactions to sounds are mediated by tactile and proprioceptive sensitivities (Piontelli, 2015). After all, the fetal ear tract is filled with fluid, and this must modulate sound propagation in specific ways that remain to be addressed (Piontelli, 2015).

In this line, studying fetuses in their own situation would bring the attention of developmental researchers toward touch. Fetal tactile experience can be observed in how the fetus explores the boundary between innervated and uninnervated regions (Mori and Kuniyoshi, 2010; Piontelli, 2015; Hata, 2016). According to these studies, fetuses frequently touch certain body areas, such as the lips, cheeks, ears, and parietal bone, creating an autostimulatory pattern, which enhances innervation. For instance, when the fetus scratches and touches the forehead, innervation increases and the boundary migrates (Piontelli, 2015). Then the fetus touches the new innervated boundary, and the cycle repeats until the whole body is fully innervated (Piontelli, 2010; Delafield-Butt and Gangopadhyay, 2013). Additionally, whether the fetus touches itself, the placenta, or a co-twin, it develops different touching patterns, that differ in pressure, acceleration and directedness (Hata, 2016). In turn, maternal touch of her own abdomen increases arm, head, and mouthing movements in the fetus (Marx and Nagy, 2015).

In phenomenology, touch has been widely explored as constituting the first and most ubiquitous perceptual experience (Lymer, 2011; Maclaren, 2014; Piontelli, 2015; Ciaunica and Crucianelli, 2019).^14^ While this might not be indicative of reflective awareness of itself or the other, we can say there is a minimal experience in the fetus of a body feeling (Ciaunica and Crucianelli, 2019). This exploration shows a particular affective dimension, as fetal touch is associated with the C-tactile afferent that regulates “affective touch”.^15^ In this sense, fetuses may have a primordial emotional life which consists in the minimal experience of pain and pleasure, comfort and discomfort, stress and relaxation. However, we should be wary of overstating the emotional capacities of fetuses (Piontelli, 2015). While some studies argue that fetal smiles, frowns or other facial expressions are indicative of an emotional life (Hata et al., 2015), we consider it misleading to infer boredom from yawns, or other complex emotional capacities that require language and reflective capacities.^16^ Nevertheless, with the coordination and integration of fetal movement and touch, we might say we are in the presence here of what phenomenologists of pregnancy characterize as intercoporeity (Gallagher, 2011; Moran, 2017; Ciaunica and Crucianelli, 2019). We will go further into this in Section “Participation.”

To recapitulate, we have seen in Section “Beginnings of Agency and Participation in Pregnancy” how mother and embryo coordinate and hold some tensions at implantation, which inaugurates the relational biological individuation process of pregnancy. This fundamental relation anchors them to the gestational process. In this section, we pointed out that this anchoring allows the fetus to keep individuating toward new forms of agency through movement and touch. The uterine environment and maternal body co-coordinate this through active-passive touching. In sensorimotor agency, we find a different way to solve the tensions of perceptual and proprioceptive experience *in utero*, which gives rise to a primitive emotional life. In the next section, we finally explore the remaining question to complete the picture: how maternal experience connects with the fetus and how the pregnant person as an agent participates in the relational process of pregnancy.

## Maternal Experience and Agency

It is time now to explore to what extent maternal bodily movement and experience contribute to and coordinate with the fetus’s developing agency. To explore the pregnant person’s agency in relation to the fetus, we connect previous empirical evidence with phenomenological insights.

### Lymer’s Maternal–Fetal Affective Communication Theory

In the introduction, we presented Lymer’s theory of affective communication as the way mother and fetus interrelate meaningfully. Now, we want to connect Lymer’s evidence for maternal–fetal affective communication with our previous discussion of sensorimotor agency. Lymer explains that the mother participates in three ways: first imprinting, then negotiating and finally affectively engaging with the fetal body schema. With this, Lymer shows that maternal sensorimotor agency participates in the emergence of fetal movement as a lived bodily experience. On top of that, we suggest, sensorimotor agency expresses a type of interaction different from that of biological agency.

Lymer (2011) starts from Merleau-Ponty’s theory of child development and his concept of the body schema. According to Merleau-Ponty (1945/2012), the body schema is our capability to integrate bodily sensations, affects, movements, and perception in such a way that we learn to move naturally without reflecting on every habitual movement we display. For instance, we grasp a glass without putting our full attention and effort into it: we do not reflect on how our arm extends, our hand opens, and our fingers grasp. Nor do we calculate the energy needed for lifting the glass. We just do it. For Merleau-Ponty (1964), the body schema emerges in the 6th month after birth.

Lymer, in contrast to this and like we did, proposes that the body schema begins developing *in utero*. She further argues that the maternal body actively participates in the development of the fetal body schema, that interactions between them create tensions that are solved by negotiating the interbodily space and, finally, that mother and fetus affectively engage. We will develop some of her claims to show how fetal sensorimotor agency interacts with maternal bodily experience. Sensorimotor schemes are one way to operationalize the phenomenological insights into the body schema (Di Paolo et al., 2017).

Within the available space in the amniotic sac, the first thing to happen, Lymer says, is that fetal movement is elicited by the mother moving her body in certain ways, e.g., walking or sitting with a particular style and rhythm. In Lymer’s words:

“[o]verall, the situation of a 10 weeks old fetus within a fluid-filled womb within a moving body amidst rhythmic beatings and breathing would facilitate a continuously moving, flowingly rhythmic world. The growing buoyant weight of the fetus at this early stage would precipitate the rolling and rocking movements that are fundamental to develop capacities for basic homoeostatic bodily positioning such as upright and sideways” (2011, p. 139).

In contrast with our account of how first fetal movements emerge from the self-organization of neural, bodily and immediate environmental aspects of the fetus, Lymer emphasizes that the fetal body schema is born from the maternal body schema. In Lymer’s account, the maternal body participates, not as merely local, biological or physical processes but as a lived bodily experience for the mother, in the specificity of her movement and the ambivalence of her affectivity.

Lymer’s reasons to defend maternal movement as the origin of the body schema are various, but we highlight two of them. First, what Merleau-Ponty (1964) calls the syncretic phase precedes the formation of the body schema and, according to Lymer, it coincides with the kind of undifferentiated movement at this stage—i.e., the fetus is moved with and by the mother. Second, Lymer rejects the widespread assumption that reflexes are the origin of fetal movement.^17^ For her, first fetal movements are regulated and practised, but reflexes are not. Besides, Lymer continues, if we take reflexes as the origin of the movement, we have to explain the developmental process of the reflex itself: How did the reflex develop in the first place? From this, she concludes that reflexes cannot be the first cause of movement; it must be maternal bodily movement.

We challenge Lymer’s argument because, as we showed in the previous section, startles are reflexes, and they set the fetal body in motion, first spontaneously and then in a self-organized manner. This does not mean that we reduce everything to reflexes, nor that we reject Lymer’s proposal. We agree that maternal movement is a necessary condition for fetal development. But we want to acknowledge both sides. On the one side, fetal-uterine interactions are locally self-organized and, on the other side, the maternal body shapes fetal movement at the global level, moving the fetus and continuously assimilating it into her body schema. Taking either maternal movement or reflexes as the single cause of fetal movement fails to recognize different levels of interaction. Global and local interactions happen at the same time—to determine which was first is like trying to answer the chicken-and-egg question. The best we can do is to fairly acknowledge both local and global aspects in the emergence of the fetal body schema.

Then, when the fetus increases in size and weight and her movements are directed with greater strength, according to Lymer, mother and fetus slowly start to participate through negotiating movement. For this, Lymer says, the fetus must show patterns of movement consistent with goal-directed action,^18^ or sensorimotor schemes according to our discussion. This happens, for instance, when a twin shows movements specifically aimed at the co-twin. This can be observed from week 14 (Piontelli, 1992; Castiello et al., 2010). On top of this, Lymer suggests that at this stage mother and fetus learn to negotiate and coordinate with each other’s movements—e.g., walking rhythmically the fetus falls asleep. For Lymer, these kinds of interactions are achieved around week 22 (Lymer, 2011).

Similar to the sensorimotor scheme in fetal suckling, habitual patterns emerge from negotiated movements and help the fetus to develop movements with greater amplitude, force, and directedness. For Lymer, this is a break-through that marks the beginning of a new level of engagement between fetus and mother. As pregnancy advances, moving involves constantly perturbing or responding to maternal flow of movement and intentions; for example, adopting a posture might be pleasurable for one, and annoying for the other. The pushes and pulls of these interactions create tensions that have to be solved by negotiating movement. Lymer vividly describes it as follows:

“As I rocked in my rocking chair in order to soothe the frustrating nocturnal movements of my fetus, the repetitive smooth rocking structured a calming synchronization between my fetus and I. Once both the movement and the affect were in line, my awareness of his presence would recede and in this example, we could then both finally fall to sleep” (Lymer, 2011, p. 132).

These negotiated movements are tinged with affective disposition. By affective, Lymer means the felt experience of the body, or how it feels to move. In the case of the mother, how it feels to move while pregnant depends on how she integrates fetal existence. Most of this occurs at the pre-reflective level, as the maternal person might experience only 16% of the total number of fetal movements (DiPietro et al., 2013 in Lymer, 2011). Affective disposition, then, is the pre-reflective way the body affectively relates to itself and the other. Lymer describes it as an affective tonality that can feel pleasant like a melody in a dance, or disruptive like an invasion. From the maternal experience, her body might feel relaxed, and her movements smooth and fluid. But as fetal movements increase in strength and their trajectories grow bigger, they will often go against her flow. Then, her body might feel stiff and tense, and her movement heavy and blundering. According to Lymer, from the moment the fetal body becomes disruptive, it becomes pre-reflectively present to the mother because her habitual body schema is not available anymore. To move fluidly again, the mother has to bring her attention to her body, meet the new effort of the task, and then she would integrate the disruptiveness and habituate to a new feeling of moving.

While Lymer does not specify in which ways the fetus also engages affectively, she mentions felt experience and touch and, as we discuss earlier, they play a role *in utero*. Similar to our point on the adultist view of development, Lymer criticizes developmental views that are too visually based and too individualistic. She also defends that touch deserves more attention, as it provides the fetus with a sense of separateness and reversibility (Lymer, 2011).

Lymer’s descriptions of maternal participation complement our account of sensorimotor agency based on the fetus and its organization. At the same time, our account of sensorimotor agency in the fetus clarifies Lymer’s point that fetal neurophysiological, bodily and sensorimotor becoming develop together with maternal movement. These processes are deeply anchored in the maternal body as a whole. Incorporating Lymer’s idea of affective communication into our account of agency brings a more sophisticated picture of maternal–fetal interaction, by showing how the mother also contributes through her lived bodily experience to the relational process of pregnancy.

### Pregnancy and Phenomenology

Understanding pregnancy as a developmental relational process can help explain how the mother as an agent is deeply and meaningfully transformed throughout. Moreover, research on the phenomenology of pregnancy has suggested that, while it can be utterly significant to it, the experience also goes beyond gender-specific female subjectivity. It pervades the very human condition, as all humans are necessarily born from women, as Adrienne Rich (1976/1995) says (see also Smith, 2016).

Iris Marion Young follows the idea that pregnancy puts into question the foundation of the unitary subject, and that pregnant persons experience and witness the ambiguity of ‘split subjectivity’:

“The first movements of the fetus produce this sense of the splitting subject; the fetus’s movements are wholly mine, completely within me, conditioning my experience and space. Only I have access to these movements from their origin, as it were. For months I can witness this life within me, and it is only under my direction of where to put their hands that others can feel these movements. I have a privileged relation to this other life, not unlike that which I have to my dreams and thoughts, which I can tell someone but which cannot be an object for both of us in the same way. Adrienne Rich reports this sense of the movements within me as mine, even though they are another’s” (Young, 2005, p. 49, referring to Rich, 1976/1995).

This externality, however, can be affectively incorporated or anchored in daily pregnant subjectivity. For Lymer (2011), maternal affective dispositions constrain and direct the formation of the fetal body schema as she incorporates the fetal body much like we incorporate artifacts into our body schema. Lymer uses the example of a person who has recently become a wheelchair user. They need to modify their body’s affective proprioception to incorporate the wheelchair and feel it as part of their body schema. In the pregnant body, however, the incorporation entails something more than a physical object. It entails the temporal adaptive accommodation of a living being. Someone who is pregnant incorporates a sensorimotor agent-in-becoming who increasingly negotiates interbodily space. Successful incorporation will depend on the affective disposition of the mother. The maternal body expresses receptiveness or resistance. It might allow the invasion to stay, scaffolding the self-individuation of the fetus, which in turn unfolds different potential agencies that might go against the maternal individuation as pregnancy advances. In their bodily relation, expanding and contracting their bodies, mother and fetus are literally modulating the scopes of their autonomies.

According to Sheets-Johnstone (1999), expansion and contraction are the basic kinetic structure by which emotion resolves itself. She describes this aspect of movement as its amplitudinal quality. For instance, the experience of our bodies as expanding and contracting can be felt in taking a deep, long breath—body and space are then felt as expanded or contracted with the movement. In Sheets-Johnstone’s own words, in this dynamic, “we are moved to move toward or against or away from” (Sheets-Johnstone, 1999, p, 267). She continues to say that in our very bodily postures, our “corporeal tonicities” make it possible to feel and to be moved to act (p. 265). In this sense, intercorporeality and its emotional load renew the primordial tensions from which agency emerges.

In the latest stages, an expansive movement of the fetus might be experienced as discomfort or even a transgression in the maternal organs, posture, and bodily movements. Indeed, according to Erwin Straus, the spatial sense of “I” that is usually located phenomenologically in our head shifts, in situations like dancing or pregnancy, from behind the eyes to the region of the trunk. Straus calls this orientation “pathic” because here we experience ourselves in greater sensory continuity with our surroundings (Straus, 1966 in Young, 2005, p. 52). This suggests a new form of self-production or “openness” accompanied by the blurred boundaries between me and the other, and between me and the world. The paradox can be put, as Lymer does, in terms of a tension between me and my pregnancy:

“Should I willingly participate in movements that facilitate a bodily synchronization then the merging of bodily movements will precipitate this blurring of boundaries and the phenomenology is an experience of being taken up or becoming caught up in the world of another. […] However, should I resist my pregnant embodiment by fighting to hold stable my pre-pregnant bodily boundaries by sustaining my previous habits then I must structure my affective engagement with the fetus as resistant” (Lymer, 2011 p. 130).

Certainly, the tensions in the felt experience are clearer to maternal experience. These maternal interactions at the global (bodily) level contrast with the picture of the local uterine environment and precisely help to distinguish that the mother has more autonomy at the global level than the fetus. Furthermore, as a person, the mother acts as biological, sensorimotor, intersubjective, and linguistic body (Di Paolo et al., 2018). In this respect, while the fetus solves tensions in bodily capacities, likeable or unlikeable sensations, the mother is endowed with additional expressive and reflective means through language, self-reflectiveness, and broader emotional and agential capacities.

### Participation

Lymer’s proposal emphasizes the affective experience of movement as a contribution from the mother to the sensorimotor agency of the fetus. We still need to better understand how interaction can create meaning for both agents through coordinated and affective movements, and how cognitive development is not simply a bilateral or symmetrical, but rather a participatory relation.

When fetus and mother step into new engagements, they enter into a new kind of relation. This emergent relation acquires a level of autonomy, and regulates their agencies, but without over-determining their autonomy. This is in line with the definition of social interaction given by De Jaegher and Di Paolo (2007), on which the enactive theory of intersubjectivity as participatory sense-making is based. But are mother and fetus engaging in participatory sense-making? We can say that pregnancy as relational process contains both of them and puts them in meaningful and affective contact. But their interactions can give rise to multiple kinds of relations, not necessarily yet intersubjective. For instance, their sensorimotor interactions can enter into phases of coordination and breakdowns, without threatening pregnancy. The foundational, biological relation between them anchors the whole process, and as such, *it* must be preserved (without it, no other relation is possible and pregnancy would stop). Pregnancy supports sense-makers in their respective developments and levels of agency and co-constitutes their agential relations with each other.

The relations and interactions that emerge during pregnancy are not static. This relates to the initial concerns regarding the metaphysics of pregnancy. The container metaphor is unsatisfactory to characterize maternal–fetal relations. They are not two already individuated systems, one inside the other. Kingma’s part-whole model begins to better account for the relational view of pregnancy, though it raises new problems. For instance: how can a self-individuating *part* and its *expansive movement* relate, and modulate its relation, with the *whole—*and, vice versa, the whole with the expansive part? On the relational-developmental view of pregnancy we have presented, both maternal organism and fetus move back and forth in the expansion and contraction of their autonomy and agency. Within the tensions that this relation entails, they can participate any time they find the conditions for interaction.

Because of this, we propose that mother and fetus participate in sense-making, minimally. This is consistent with our analysis because the way each of them participates in their relation—as the agents they are at each stage—transforms them in meaningful ways. Note that they are not constituting, but modifying each other’s individuation processes. Thus, from this point of view, their perspective on the world is bodily and affectively intertwined in their interaction. As every interaction changes their situation in the world, the way they sense, make sense of, and value the world also changes. As such, the relation between mother and fetus can be considered at least minimally intersubjective. First experiences in the fetus are already confronted with the mother’s alterity in minimal ways, both locally, as a moving and rhythmic world; and globally (and later in development), when mother and fetus engage as two agents, from their own perspectives. Within the theory of intersubjectivity as participatory sense-making (De Jaegher and Di Paolo, 2007; Fuchs and De Jaegher, 2009; De Jaegher, 2018), even these (asymmetrical) forms of intercorporeality form an initial part of an explanation of intersubjectivity.

## Conclusion: Not One, Not Two

In this paper, we have raised the question of agency in mother and fetus, and of the interactions between them. It is not possible to provide a fully satisfactory answer to the problem based on an abstract, analytical framework that looks at how parts relate to whole, or whether the fetus is merely contained in an environment. We have found that we must understand how mother and fetus are pulling apart from each other, and still maintain an immediate relation that emerges as an autonomous relational organization. Physically, there is immediate contact between the fetus and the amniotic fluid. But at the same time, organizationally, there is a mediation between them, beginning when at some point the fluid starts to move through the body of the fetus, and later it will be moved by the fetus itself. We arrived here by changing the way we look at pregnancy, from the agents to their relations and back again. For this, the enactive concepts of self-distinction and self-production have proven helpful, and will still be needed to further clarify issues that we have not addressed here. A relational, processual view of becoming, individuating agents rather than static entities (individuals) thus changes our understanding of agency and autonomy.

In the same spirit, looking at the phenomenon as deeply relational, embodied and processual will change our conception of pregnancy. First, it acknowledges the active role of the pregnant person during gestation. In the social context, this can contribute to changing the perception of pregnancy as a passive and weak condition, and go toward a more robust and active idea of female bodies in general. Even so, as we suggested here, pregnancy is a hugely transgressive process and as such women require the most caring, responsive and supportive environment possible, whether in economic, affective, social, or institutional terms. For the fetus, this implies recognizing the great adaptive capacities that it develops during pregnancy to survive radical changes *in utero* and the dramatic environmental transition it has to accommodate upon and after birth. With these insights, we expect cognitive science to continue studying fetal development in its own right, without assuming the neonate (or adult) as the reference point. Furthermore, taking the enactive stance, this means for cognitive science and developmental studies that sense-making can be studied by looking at the interaction between fetus and mother, as an interaction to which they both relate, in a dialectical move that follows Varela’s (1976) idea of “not one, not two.” Even further, it means that what fetus and mother are doing during pregnancy is generating meaning in their intercorporeal interacting. All of these aspects have to be further explored. We consider we have provided enough elements to open up these questions for future empirical research.

Last but not least, our proposal can be read in relation to the question of abortion. While this is not the topic of this paper, we can make a few remarks on it. First, agency in cognitive science must not be understood as an arbitrary property, either by political convenience or moral convention. It must be understood as a phenomenon that emerges from the system’s mode of operation. In this sense, even if the system can fail or be alienated, the agency might remain as the potential capability to modulate at least some of its interactions with the world in some moments. We have used and developed the concepts of agency and autonomy here in this technical sense. This can serve to refine ethico-political discussions. Second, attributing agency to an organism, even in a complex form, does not imply that this organism is a human being. There is a large literature that studies unicellular and multicellular activity as agency (bacteria, plants, and animals), and nevertheless, they are not necessarily subject to the same ethical considerations as persons are—although maybe in some cases they should be. And third, the moral or political dimensions of agency, especially when talking about abortion, require a wider elaboration of humanity and life’s dignity that we did not address here. For instance, the recognition of suffering in fetus and pregnant person; issues of dignity or advisable death; or why some living forms should or should not be taken into consideration (e.g., human vs. non-human, fetus vs. mother). In this line, we do have a political stance: maternal persons should never be obliged to undergo such a transgressive process against their will, to the detriment of both fetal and maternal quality of life. These issues are beyond the scope of this piece, but other works address more specifically this question of political agency in relation to pregnancy (Rich, 1976/1995; Young, 2005; Lymer, 2016; Chadwick, 2018; Lewis, 2019). We encourage others to use the enactive elaboration here provided to connect with these political concerns in future work. At the moment, our argumentation about the relational aspects of pregnancy is far from attributing rational or moral capacities to the fetus. Instead, we propose a new way to look at pregnancy and the way it anchors, holds and co-determines the *beginnings* of human-like forms of agency and participation.

## Author Contributions

AM came up with the original idea (in part based on discussions with Michaela Pavličev, Laura Nuño de la Rosa, and Arantza Etxeberria, with whom we co-organised the Forgotten Female Bodies Workshop, Universidad del País Vasco, San Sebastián, 2018). AM researched the biological and phenomenological literatures on pregnancy. AM and HD together worked out the enactive argument and wrote the manuscript, and both approved the final version.

## Conflict of Interest

The authors declare that the research was conducted in the absence of any commercial or financial relationships that could be construed as a potential conflict of interest.

## Footnotes

1 One starting point of the enactive approach—which we take in this paper, as we explain in a moment—is that “life can be known only by life,” as Hans Jonas says (1966, see also Weber and Varela, 2002; Di Paolo, 2005; Thompson, 2007; Di Paolo et al., 2010). This means that, as living beings, we can recognize the stakes living beings have in their life. The idea is in line with existentially inclined scientists like Helmut Plessner, Frederik J. J. Buytendijk, Kurt Goldstein, Michael Polanyi, Georges Canguilhem, Erwin Strauss (Di Paolo, 2005). This entails a particular epistemological approach, which we avail ourselves of, but do not as such defend here. De Jaegher (2019) sets it out.

2 This paper is about human pregnancy (not: pregnancy in non-human animals). In our view, the question of which interactions are at play during pregnancy can benefit from a phenomenological analysis, i.e., looking into human experience. Thus, throughout the paper, we will refer to human experience by referring to the maternal organism or the mother, and we will use uterine or maternal environment to refer to the non-experiential or localized contributions of the maternal body. We interchangeably use the terms maternal organism, maternal body, mother, and pregnant person to refer to the one who is pregnant, assuming—in general—a female body, but also acknowledging the diversity of humans who can be pregnant, including trans men and non-binary people (by also using “person” in places). [As we will see later (footnote 9), we do not use “pregnant body” for a gender-neutral term, because we have a different theoretical use in mind for “pregnant body”].

3 Maturana and Varela (1980) described the boundary as a physical division, as the production of a cell membrane, as a condition for the existence of the internal chemical network. But not all boundaries have to be physically constituted: individuation is the process of differentiating something both functionally and spatially.

4 The discussion about egg and zygote being autopoietic systems needs further elaboration but exceeds the scope of this paper. However, with the evidence available so far, we propose that oocytes can be tentatively characterized as autopoietic systems.

5 Note that for Nuño de la Rosa (2010), the morula is not an individual, because blastomeres are not functionally and structurally integrated.

6 In fact, these are not clinically recognized as pregnancies (Norwitz et al., 2001).

7 With thanks to one of the reviewers, who insisted on this point and provided this expression.

8 Partially at least, because the maternal body will provide for metabolic demands and regulate gene expression to a great extent throughout pregnancy. Agency is not all or nothing but can be a transient power of the living system. It is not always fully reached. Sometimes the fetus has agency, and it is necessary, we argue, for certain developmental transitions.

9 A further proposal for characterizing pregnancy in this line is to conceptualize this relational organization as a *pregnant body*, in analogy with the theory of bodies introduced in *Linguistic Bodies* (Di Paolo et al., 2018). Working out this idea is outside the scope of this paper, but it can be a fruitful basis for further research. It is the notion of dialectical defined in this book (Di Paolo et al., 2018, chapter 6) that we also use here. Describing pregnancy as a dialectic, we do not mean it is a situation of balance and equilibrium that takes already constituted entities as premises. Rather, we understand it as involving metastability, tensions, and constituting relations. Transformations of tensions in one stage lead not to resolutions (or synthesis), but to new tensions in the next stage.

10 Di Paolo et al.(2017, p. 43) describe sensorimotor coordination as “organized in the context of a task,” but the notion of “task” is not applicable in the same way in—what are often—laboratory studies and *in utero*. However, we will assert, and will also see this again in Section “Lymer’s Maternal–Fetal Affective Communication Theory,” that there is a certain regulation and goal-directedness to sensorimotor coordination in the fetus’s movements, when seen in their context.

11 The central pattern generator is a neural network that produces the rhythmic patterns of activity, like breathing movements. These movements are produced in the absence of any rhythmical input. For instance, breathing movements are independent of maternal breathing (Piontelli, 2010; ten Donkelaar et al., 2014).

12 The beginning of swallowing movements also coincides with intestinal loops coming back into place, after a natural prolapse (Trahair, 2001, p. 139).

13 Another example of this is the controversial question of neonatal imitation (see, e.g., Meltzoff and Moore, 1977; Gallagher and Meltzoff, 1996; Oostenbroek et al., 2016; Vincini et al., 2017; Meltzoff et al., 2018). In this case, our analysis suggests it should be fruitful to investigate whether newborns have already practised the relevant movements *in utero*. In fact, some ultrasound studies suggest that most movements observed in neonatal life can already be identified in fetuses (Stanojevic et al., 2011). What we observe as imitation may therefore be the neonate’s efforts to accommodate and assimilate the new environmental conditions into previous schemes.

14 With thanks to one of the reviewers, who suggested to highlight aspects of touch.

15 In the fetus, touch is mediated by the C-Tactile afferent found in the lanugo—the hairy skin of the fetal body; and C-Tactile afferents are the kind of neurons that innervate the human skin, and regulate pain perception and so-called “affective-touch,” which is slow in acceleration and low in pressure (Piontelli, 2015; Ciaunica and Crucianelli, 2019).

16 We cannot elaborate on this point, but it would be an interesting question for future research.

17 Like Thelen and Smith (1994), Lymer rejects the tendency in some developmental research to see reflexes as the origin of movement and to reduce explanation to a neural basis.

18 As shown by the KANET score (Kurjak et al., 2008). Using dynamical systems methods, this score can determine the level of directedness or regulatory movements in fetuses. The test classifies abrupt, cramped, non-fluent, small range movement as less or non-regulated, while smooth, fluid and complex movements with a full range might suggest more regulation (Hata, 2016).
